# INCISIONAL HERNIOPLASTY TECHNIQUES: ANALYSIS AFTER OPEN BARIATRIC SURGERY

**DOI:** 10.1590/0102-672020200002e1517

**Published:** 2020-11-20

**Authors:** André Thá NASSIF, Thais Ayumi NAGANO, Izabela Rodrigues VILLELA, Giulianna Ribas SIMONETTI, Bruno Francisco DIAS, Alexandre Coutinho Teixeira de FREITAS

**Affiliations:** 1 Digestive and Bariatric Surgery Service, Santa Casa de Misericórdia, Curitiba, PR, Brazil; 2 Postgraduation Program in Surgical Clinics, Federal University of Paraná, Curitiba, PR, Brazil; 3 Medical Degree, Pontifícia Universidade Católica do Paraná, Curitiba, PR, Brazil

**Keywords:** Incisional hernia, Bariatric surgery, Hernia, ventral, Hérnia incisional, Cirurgia bariátrica, Hérnia, ventral

## Abstract

**Background:**

The best technique for incisional hernioplasty has not been established yet. One of the difficulties to compare these techniques is heterogeneity in the profile of the patients evaluated.

**Aim:**

To analyze the results of three techniques for incisional hernioplasty after open bariatric surgery.

**Method:**

Patients who underwent incisional hernioplasty were divided into three groups: onlay technique, simple suture and retromuscular technique. Results and quality of life after repair using Carolina’s Comfort Scale were evaluated through analysis of medical records, telephone contact and elective appointments.

**Results:**

363 surgical reports were analyzed and 263 were included: onlay technique (n=89), simple suture (n=100), retromuscular technique (n=74). The epidemiological profile of patients was similar between groups. The onlay technique showed higher seroma rates (28.89%) and used a surgical drain more frequently (55.56%). The simple suture technique required longer hospital stay (2.86 days). The quality of life score was worse for the retromuscular technique (8.43) in relation to the onlay technique (4.7) and the simple suture (2.34), especially because of complaints of chronic pain. There was no difference in short-term recurrence.

**Conclusion:**

The retromuscular technique showed a worse quality of life than the other techniques in a homogeneous group of patients. The three groups showed no difference in terms of short-term hernia recurrence.

## Graphical Abstract

[Fig f1a]

**Figure f1a:**
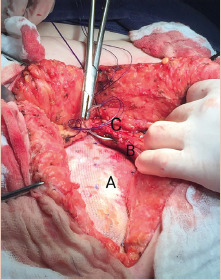
Rives-Stoppa retromuscular technique: A) polypropylene mesh fixed on the posterior rectus sheath; B) rectus abdominal muscle; C) anterior rectus sheath being sutured

## RESUMO

**Racional:**

A melhor técnica de hernioplastia incisional ainda não foi definida. Uma das dificuldades na comparação é a heterogenicidade no perfil dos pacientes avaliados.

**Objetivo:**

Analisar os resultados de três técnicas de hernioplastia incisional após cirurgia bariátrica aberta.

**Método:**

Os pacientes que realizaram hernioplastias incisionais foram estudados e divididos em três grupos: técnica onlay, sutura simples e técnica retromuscular. Resultados e qualidade de vida após o reparo utilizando o Carolina’s Comfort Scale foram avaliados através de análise de prontuários, contato telefônico e consultas eletivas.

**Resultados:**

Foram analisados 363 relatos cirúrgicos e 263 foram incluídos: técnica onlay (n=89), sutura simples (n=100), técnica retromuscular (n=74). O perfil epidemiológico dos pacientes foi similar entre os grupos. A técnica onlay apresentou maiores taxas de seroma (28,89%) e usou dreno com maior frequência (55,56%). A sutura simples obteve maior tempo de internação (2,86 dias). O escore de qualidade de vida foi pior na técnica retromuscular (8,43) em relação à onlay (4,7) e à sutura simples (2,34), especialmente devido às queixas de dor crônica. Não houve diferença quanto a recidiva em curto prazo.

**Conclusão:**

A técnica retromuscular apresentou pior qualidade de vida do que as demais técnicas em um grupo homogêneo de pacientes. Os três grupos não mostraram diferenças em termos de recidiva de hérnia em curto prazo*.*

## Graphical Abstract

[Fig f1as]

**Figure f1as:** Técnica retromuscular de Rives-Stoppa: tela de polipropileno fixada no folheto posterior do reto; B) músculo reto do abdome; C) folheto anterior do reto sendo suturado.

## INTRODUCTION

One of the most frequent late complications of laparotomies is incisional hernia, which occurs between 11-23% of patients, but it can reach up to 50% in ones with high-risk ^1^. Risk factors known for making up incisional hernia include male gender, advanced age, obesity, previous abdominal surgery, smoking, chronic obstructive pulmonary disease, and others^1^ ^,^ ^10^ ^,^ ^23^. Invariably, patients with indication for bariatric surgery have multiple of such risk factors.^1^ ^,^ ^18^ ^,^ ^23^ The incidence of incisional hernia after open Roux-en-Y gastric bypass (RYGB) varies between 8-20%^19^.

The treatment of incisional hernia is essentially surgical and it basically involves: identification of the hernia sac, reduction of the content and closure of the defect. Most patients will need a procedure that uses tension-free repair with prosthetic reinforcement^12^. Among the possible techniques used for repair of incisional hernias are the onlay technique (OT), the simple suture (SS) and the retromuscular technique (RMT).

OT, also called pre-aponeurotic, is one of the most popular techniques among surgeons, as it is fast and effective. The mesh is fixed on the abdominal wall defect, above the anterior rectus sheath^7^ ^,^ ^12^ ^,^ ^22^.

SS without mesh was the standard treatment until the 1990s. Due to the high rate of recurrence, most studies recommend abandoning this technique in defects larger than 5 cm^3,^ ^20^. 

RMT was described by Rives-Stoppa and it consists of dissection between the rectus muscle and the posterior rectus sheath to allow the placement of a sublay mesh. Some authors advocate that this procedure should be standard means of comparison for other techniques, especially in complex incisional hernias^5^ ^,^ ^7^.

Among mesh repairs, there is still controversy as to which one is the best, especially due to lack of studies with proper methodological aproach^21^ ^,^ ^22^.

The aim of this study was to compare the results of three different hernioplasty techniques, using a homogeneous group of incisional hernias from exclusively open bariatric surgery.

## METHOD

Patients who underwent incisional hernioplasties from January 2015 to December 2016 were analyzed, including only hernias from open bariatric surgery, either RYGB or sleeve gastrectomy (SG). All the surgical procedures were performed at Hospital Santa Casa de Misericórdia in Curitiba, PR, Brazil. The patients signed the Free and Informed Consent Form before undergoing the two operations. This study was submitted to the Research Ethics Committee and approved on October 30, 2017, CAAE n. 72098417.8.0000.0020. Data were initially collected through medical records. The follow-up of patients was carried out prospectively through phone calls and medical appointments at the hospital’s facilities.

All patients were obese or ex-obese, had a supraumbilical median laparotomy scar ranging from 12-15 cm and had hernias classified as M2, W2 or 3 according to the European Hernia Society^17^.

Patients who did not respond to the telephone call or did not attend the medical appointment were kept in the survey, and in such cases only the data found in the medical record were used.

Before the start of the study, these patients were randomly referred for surgery by the general surgeons of the hospital, following the normal flow of the surgical schedule. Regardless of the size of hernia or other characteristics of the abdominal wall, one of the surgeons performed the SS exclusively, another performed the RMT routinely, and all the others performed the OT. Thus, after the medical records were analyzed, these patients were divided into three groups according to the technique used to repair incisional hernias: group A - OT; group B - SS; group C - RMT. All surgical descriptions were reviewed to check what technique has been performed. Patient´s exclusion criteria were as follows: with incisional hernia from other operations; with bariatric surgery performed in other hospitals or via laparoscopic surgery; those who had undergone any other hernioplasty technique; and patients who did not agree to participate in the study.

In group A, OT consisted of identifying the defect and dissecting the anterior rectus sheath of the subcutaneous tissue. The defect was closed by means of a continuous suture using 1 polydioxanone, and a polypropylene mesh was fixed with simple 2-0 polypropylene stitches.

In group B, SS was performed by identifying the anterior rectus sheath and closing the hernia defect in three suture planes, two of which are continuous 1 polydioxanone and one with X points of 1 polydioxanone.

**FIGURE 1:**
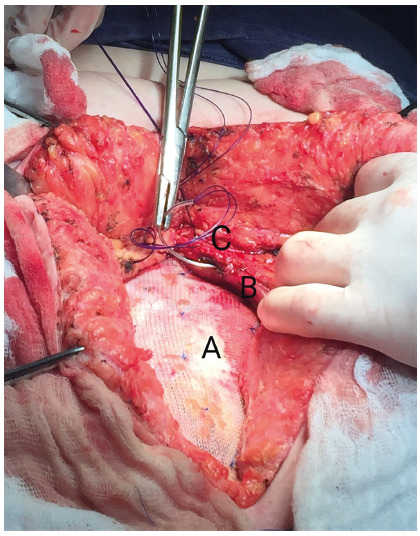
Rives-Stoppa retromuscular technique: A) polypropylene mesh fixed on the posterior rectus sheath; B) rectus abdominal muscle; C) anterior rectus sheath being sutured.

In group C, RMT started with the identification of the defect and resection of the hernia sac. The posterior rectus sheath was dissected from the rectus muscle to the semilunar line. The posterior rectus sheath was closed with a continuous 1 polydioxanone suture, and the polypropylene mesh was fixed above this plane with simple 3-0 polypropylene stitches (Figure 1). Finally, the anterior rectus sheath was also closed with a continuous 1 polydioxanone suture to avoid contact of the mesh with the subcutaneous tissue.

To test whether the groups were homogeneous, patients were initially compared as to gender, age, time between bariatric operations and hernia diagnosis, bariatric surgery technique, weight loss, comorbidities and other previous operations.

The groups were also compared regarding the use of a surgical drain, the performance of a simultaneous procedure to repair the hernia, the length of hospitalization stay, the length of stay in the intensive care unit, surgical wound complications (seroma, hematoma and infection), new hospitalizations, reoperations, quality of life (QOL) after hernia surgery and hernia recurrence rate.

Regardless of the hernioplasty technique used, the possible risk factors for complications such as BMI, excess weight loss (EWL) or total weight loss (TWL), type 2 diabetes mellitus, smoking, previous surgery at upper abdomen and recurrent hernia were compared with surgical wound complications (seroma, hematoma and infection), new hospitalization, reoperation, and QOL after hernia surgery and hernia recurrence rate.

Another comparison was made between the surgical wound complications (seroma, hematoma and infection), new hospitalization, reoperation and hernia recurrence rate, with the final QOL score, in order to verify the impact of these factors.

Carolina’s Comfort Score (CCS) was used for QOL analysis. The questionnaire applied by phone call quantifies three symptoms (pain, mesh sensation and limitation of movement) during the performance of eight activities: lying down, when bending, sitting, doing activities of daily living, when coughing or breathing deeply, when walking, when climbing stairs and when performing physical exercise. Each response ranges from the absence of symptoms (0) to disabling symptoms (5) and thus the total score is between 0 and 115. Each symptom score, during each activity, was also analyzed separately and thus, a 0 score was considered absence of symptoms and a score of 1 as symptomatic. Likewise, a total score equal to 0 was considered totally asymptomatic^2^ ^,^ ^9^.

During the telephone call, all data obtained from the analysis of medical records were confirmed and the medical appointment was scheduled. In each call, a physical examination was performed looking for hernia recurrence and the Informed Consent Term was applied.

Recurrence was defined as all cases that presented herniation on physical examination by the time of the appointment, performed hernia reoperation due to recurrence or documented evolution of recurrence in the chart through physical examination or image examination.

### Statistical analysis

The data were recorded on Google Sheets^®^ and transferred to Excel^®^ for statistical analysis. The QuiSquare test was used for categorical variables and the Kruskal-Wallis test was used for continuous variables. Statistical significance was defined as p <0.05.

## RESULTS

Three hundred and sixty-three surgical reports of hernioplasty were identified. Out of them, 263 resulted from bariatric surgery. There were 243 patients and 20 reoperations: 89 patients in group A (OT), 100 in group B (SS) and 74 in group C (RMT).

Of the total number mentioned above, 167 (68.7%) answered the phone call and 157 (64.3%) by questionnaire. A total of 101 (41.5%) patients attended the scheduled appointment and were examined.

Most patients (91.36%) were female and the average age was 45.55 years. RYGB was the most used bariatric surgery technique (87.6%) and the previous average BMI was 42.93 kg/m². The average time interval between bariatric surgery and hernia repair was 464.45 days, the average BMI during the repair was 29.39 kg/m², %EWL was 76.32% and %TWL was 31.80% (Table 1).

There was no difference between BMI, %EWL, %TWL, diabetes, smoking and other previous operation in the upper abdomen between the groups. Group C had more hypertension and SG as a bariatric procedure, but as a whole the groups were homogeneous (Table 1).

Table 2 shows the collected perioperative data. Group B had more simultaneous procedures (55.45%), such as omentectomy for visceral reduction, cholecystectomy or tactical appendectomy. Group A used more drainage (55.56%) than the other groups, mainly with tubular drainage. Hospital stay was longer in group B (2.86 days) than in group A (2.41 days) and C (2.22 days), but there was no difference between the groups as to length of stay in the intensive care unit.

**TABLE 1:** General data

Variables	Group A	Group B	Group C	Total	p
Gender					0.121
Female	81 (95.29%)	87 (91.58%)	54 (85.71%)	222 (91.36%)	
Male	4 (4.71%)	8 (8.42%)	9 (14.29%)	21 (8.64%)	
Total	85	95	63	243	
Age (average)					0.15
During hernioplasty (SD)	46.58 (8.17)	44.51 (8.91)	45.73 (9.8)	45.55	
Bariatric surgery or reoperation					0.021¹
RYGB	78 (91.76%)	86 (90.53%)	49 (77.78%)	213 (87.65%)	
SG	7 (8.24%)	9 (9.47%)	14 (22.22%)	30 (12.35%)	
Reoperation of hernioplasty	5	6	9	20	
Total	90	101	72	263	
Percentage EWL					0.081
During hernioplasty (SD)	78.48 (17.64)	76.96 (16.23)	72.88 (16.5)	76.32 (16.87)	
Previous hypertension					0.024²
Yes	46 (54.76%)	57 (60.00%)	48 (76.19%)	151 (62.40%)	
Total	84	95	63	242	
Previous diabetes					0.1
Yes	14 (16.67%)	23 (24.21%)	20 (31.75%)	57 (23.55%)	
Total	84	95	63	242	
Previous smoking					0.35
Yes	15 (17.86%)	15 (15.79%)	6 (9.52%)	36 (14.88%)	
Total	84	95	63	242	
Presence of hernia prior to bariatric surgery					0.44
Yes	3 (3.33%)	2 (1.98%)	4 (5.56%)	9 (3.42%)	
Total	90	101	72	263	
Other previous operation in the upper abdomen					0.083
Yes	21 (23.60%)	17(16.83%)	22 (31.43%)	60 (23.08%)	
Total	89	101	70	260	
Recurrent hernia					0.18
Yes	6 (6.74%)	10 (9.90%)	11 (15.71%)	27 (10.38%)	
Total	89	101	70	260	

¹=technique with significantly higher percentage of patients that had sleeve as previous bariatric surgery; ²=technique with significantly higher percentage of patients with previous hypertension; RYGB=Roux-en-Y gastric bypass; SG=sleeve gastrectomy; SD=standard deviation

**TABLE 2:** Perioperative data

Perioperative data	A	B	C	p
Simultaneous procedures	12.22%	55.45%	13.89%	0.00001
Drain use	55.56%	21.78%	37.5%	0.00001
Hospital stay	2.41 days (SD 0.67)	2.86 days (SD 1.14)	2.22 (SD 0.54)	0.00001
ICU* stay	0 (SD 0)	0.12 day (SD 0.53)	0.06 day (SD 0.29)	0.072

ICU=intensive care unit

Regarding complications (Table 3), group A had higher rates of seroma than the other groups (A=28.89%, B=10.89% and C=9.72%; p=0.00069) and a higher rate of surgical site infection (SSI) when compared to group B (A=22.22%, B=9.9%; p=0.0195). There was no difference in the rate of hematoma. The rate of hospital readmission was around 6% and the rate of emergency reoperation was around 4%. There was no difference between the three groups. 

Hernia recurrence was detected in 38 cases: 15 (16.67%) in group A, 16 (15.84%) in group B and 7 (9.72%) in group C. There was no statistical difference between the three groups (p=0.409). The average time between the operation and the medical appointment was 784 days.

**TABLE 3:** Complications after hernioplasty

Complication	A	B	C	Total	p	Complement
Seroma	26 (28.89%)	11 (10.89%)	7 (9.72%)	44	0.00069	A≠B (p=0.0017)
						A≠C (p=0.0026)
Hematoma	9 (10.00%)	8 (7.92%)	5 (6.94%)	22	0.767	--
SSI	20 (22.22%)	10 (9.90%)	13 (18.06%)	43	0.064	A≠B (p=0.0195)
Recurrence	15 (16.67%)	16 (15.84%)	7 (9.72%)	38	0.403	--
Hospital reentry	6 (6.67%)	7 (6.93%)	4 (5.56%)	17	0.932	--
Urgency reoperation	4 (4.44%)	4 (3.96%)	3 (4.17%)	11	0.986	--
Elective recurrent hernia reoperation	10 (11.11%)	10 (9.90%)	3 (4.17%)	23	0.26	--

SSI=surgical site infection

The total CCS score is shown in Table 4. Group A had an average of 4.7; group B 2.34; and group C 8.43 (p=0.0028). Group C had CCS scores significantly higher than group B (p=0.0009). The number of totally asymptomatic patients (score 0) was lower in group C (A=60.38%, B=72.22%, C=46.81%; p=0.013).

**TABLE 4:** Carolina’s Comfort Scale (CCS) total comparative score

Variables	Group A	Group B	Group C	p
Average of CCS	4.7	2.34	8.43	0.0028¹
SD	9.46	6.45	14.1	
Number of 0 scores	32 (60.38%)	65 (72.22%)	22 (46.81%)	0.013²
Maximum score	43	36	61	

¹p=value between B and C: 0.0009; ²p=value between B and C: 0.006; SD=standard deviation

Comparing the presence of symptoms in each CCS question (Table 5), group A obtained lower scores than group C when patients were asked about the presence of pain when lying down (A=1.89%, C=13.04%; p=0.0369) and when bending (A=13.21%, C=30.43%; p=0.0365). Pain when exercising remained less frequent in group A than in group C (A=11.32%, C=23.26%; p=0.0328) but it was also less frequent in group A when compared to group B (A=11.32%, B=31.03%; p=0.0117).

**TABLE 5:** Comparison of the presence of symptoms on each Carolina Comfort Scale (CCS) question after hernioplasty

CCS question	Group A		Group B		Group C		Analysis	
	Yes	%	Yes	%	Yes	%	p	Complement
Lying down- mesh?	8	15.69%	0		9	20.00%	0.58*	--
Lying down - pain?	1	1.89%	3	5.17%	6	13.04%	0.0685	A≠C (p=0,0369)
Bending over - mesh?	7	13.73%	0		10	22.22%	0.28*	--
Bending over - pain?	7	13.21%	12	20.69%	14	30.43%	0.11	A≠C (p=0,0365)
Bending over - limitation?	5	9.43%	8	13.79%	6	13.04%	0.76	--
Sitting up - mesh?	2	3.92%	0		2	4.44%	0.90*	--
Sitting up - pain?	2	3.77%	4	6.90%	5	10.87%	0.39	--
Sitting up - limitation?	0	0.00%	1	1.72%	0	0.00%	0.42	--
Daily living - mesh?	5	9.80%	0		6	13.33%	0.59*	--
Daily living - pain?	7	13.21%	8	13.79%	8	17.39%	0.82	--
Daily living - limitation?	4	7.55%	6	10.34%	4	8.70%	0.87	--
Cough/breath - mesh?	5	9.80%	0		3	6.67%	0.58*	--
Cough/breath - pain?	5	9.43%	3	5.17%	5	10.87%	0.54	--
Cough/breath - limitation?	2	3.77%	0	0.00%	3	6.52%	0.16	--
Walk/stand - mesh?	2	3.92%	0		3	6.67%	0.55*	--
Walk/Stand - pain?	3	5.66%	5	8.62%	6	13.04%	0.44	--
Walk/stand - limitation?	2	3.77%	0	0.00%	3	6.52%	0.16	--
Stairs - mesh?	3	5.88%	0		3	6.67%	0.87*	--
Stairs - pain?	4	7.55%	7	12.07%	8	17.39%	0.32	--
Stairs - limitation?	1	1.89%	6	10.34%	2	4.35%	0.14	--
Exercising - mesh?	5	9.80%	0		6	13.33%	0.59*	--
Exercising - pain?	6	11.32%	18	31.03%	13	28.26%	0.034	A≠B (p=0,0117) A≠C (p=0,0328)
Exercising - limitation?	2	3.77%	7	12.07%	5	10.87%	0.26	--

*=only two groups were compared (A and C)

Regardless of the hernioplasty technique, patients with previous hernia repair had a higher rate of SSI (44.44% vs. 15.35%; p=0.04). No statistical significance was found between BMI at the time of hernioplasty, %EWL or %TWL, diabetes and smoking in relation to surgical wound complications (seroma, hematoma and infection), new hospitalization, reoperation, QOL after hernia surgery and rate of hernia recurrence.

Patients who scored above 0 in the CCS had higher rates of SSI (22.54% vs. 11.76%; p=0.049). Seroma, hematoma, new hospitalization, reoperation and hernia recurrence did not statistically influence the CCS (Table 6).

**TABLE 6:** Comparison of complication rates between the groups of patients scoring 0 in the Carolina Comfort Scale (CCS) and the group with CCS different from 0 after hernioplasty

Complications	CCS 0	CCS >0	p
Seroma	12.61%	19.72%	0.19
Hematoma	6.72%	8.45%	0.66
SSI	11.76%	22.54%	0.049
Recurrence	6.72%	12.68%	0.16
New hospitalization	5.04%	7.04%	0.39
Emergency reoperation	4.20%	4.23%	0.63
Elective recurrent hernia reoperation	4.20%	1.41%	0.27

SSI = surgical site infection

## DISCUSSION

There is still no consensus on the best technique for incisional hernioplasty, and surgeons generally rely on their own experience, not on clinical evidence^20^.

The major difficulties in comparing these surgical techniques arise from the heterogeneity in the profile of patients, the differences in size and complexity of the hernias to be corrected and the lack of standardization in the approach to obtain results after the hernioplasty is performed^16^ ^,^ ^22^. Recently, pain assessment and postoperative QOL have become important measures for evaluating the outcome after surgery^2^ ^,^ ^9^ ^,^ ^13^.

In this study, we compared three techniques used to treat incisional hernias originating exclusively from a standard open bariatric surgery incision, either RYGB or SG.

Objective results and QOL after surgery were analyzed. The compared results were rates of seroma, hematoma, SSI, recurrence, hospital readmission, urgent and elective reoperation. The QOL of patients was assessed using the CCS score, validated in 2007, used in more than 40 countries and accessible online or by email. As it is a specific score for the evaluation of patients who undergo hernia repairs using mesh, CCS is considered more efficient than other generic QOL questionnaires such as the Short-Form 36^9^ ^,^ ^16^ ^,^ ^23^.

The general profile of the patients was as expected for a population that undergoes bariatric surgery through the Brazilian Unified National Health System (SUS): 91.36% female, average age was 45.55 years and average prior BMI was 42.93 kg/m². The low proportion of SG (12.35%) compared to RYGB is also found at SUS^14^ ^,^ ^15^.

The average time interval between bariatric surgery and hernia repair of 464.45 days can be explained by representing the average time that is expected for maximum weight loss. This is the probable reason why the average BMI during the correction was 29.39 kg/m² and the weight loss was satisfactory with %EWL of 76.32% and %TWL of 31.80%. 

By analyzing the comorbidities present before bariatric surgery, it can be observed that 62.4% were hypertensive, 23.5% were diabetic and 14.8% were smokers. It can be said that it was a population at risk for developing hernia and complications in general. In addition, 23% of the patients had other previous surgery in the upper abdomen and 10% of the operated hernias were already recurrent, predictive factors of greater technical difficulty.

The three groups in this study were homogeneous in terms of age, time between bariatric surgery and hernia diagnosis, weight loss, diabetes, smoking, other previous operations and recurrent hernia. RMT patients had a higher prevalence of hypertension and a higher proportion of SG as a technique used in bariatric surgery, but this was not considered relevant for a worse outcome after hernioplasty.

OT presented the highest seroma rates (22.8%). This fact has also been observed in many studies and it is easily explained by the creation of a huge dead space between the anterior rectus sheath and the subcutaneous tissue. The literature considers seroma as a minor complication that usually presents a good outcome. Due to the high incidence of seromas, some studies recommend the use of a suction drain, which was adopted in this group in 55% of patients^9^ ^,^ ^22^.

OT presented higher rate of SSI than SS. The use of a mesh is the probable reason for this difference^20^.

The higher rate of hospitalization in group B and the higher proportion of simultaneous procedures can be explained by the particularities of the surgeon who performed SS.

Justified by the complexity of the cases, the general rate of readmission was 6% and the rate of emergency reoperation was 4%. The vast majority of reoperations were due to surgical wound complications such as drainage of larger volume hematomas and abscesses. This piece of data is rarely documented in the largest series, but it can occur from 0-6%^4^ ^,^ ^8^ ^,^ ^12^.

The total CCS values of the SS were the lowest. This piece of data should be interpreted with caution as the CCS is more specific for hernia repairs with mesh and patients in this group always score 0 on mesh sensation questions, thus lowering the total score^11^. By detailing each complaint, SS patients had more pain symptoms when exercising than did OT patients. Burger et al^4^ documented more severe abdominal pain in patients who underwent SS compared to those who underwent mesh, probably due to the difference in tension in the wound.

RMT obtained the highest total CCS values and presented the symptom of pain when lying down, when bending over and when doing physical exercises more often than OT did. Comparisons between post-operative CCS of RMT, OT and other component separation techniques have been made in some studies, but there was no difference between groups. However, there is a tendency for CCS values to improve over time (up to one year), but further studies are needed for long-term verification^2,13.^

Chevrel and Rath^6^ compared the recurrence rates of OT and SS, and they obtained results of 9.02% vs. 18%, respectively. Contrary to the literature, there was no higher rate of hernia recurrence in SS when comparing the techniques that used mesh (A=16.67%, B=15.84% and C=9.72%; p=0.409)^20^. For patients with large weight losses, closure of the abdominal wall may naturally present less tension, which could explain the non-increase in recurrence rate.

This study has some limitations that should be highlighted. The size of the hernia defect was not objectively assessed and therefore it cannot be ruled out that there is a difference between the groups, though that’s unlikely. The ideal follow-up would be at least five years and in this study it ranged only from one to three with an average of just over two years. In addition, the small proportion of patients who returned to the postoperative clinical evaluation (41.5%) impaired the clinical diagnosis of herniated recurrences not documented in medical records. For these reasons, the assessment of recurrence was not the main focus of this article, though it could be better evaluated in a future study.

Our results can guide an individualized decision for each patient. We recommend selecting the technique according to the patient’s profile, hernia size and abdominal wall condition. OT is a good technique for simple abdominal wall defects, but it leads to more surgical wound complications. RTM can be indicated in complex cases, but not routinely, as it can worsen QOL.

## CONCLUSION

This study demonstrated differences in the results of the three techniques used to correct incisional hernias resulting from open bariatric surgery. RMT had a worse quality of life, especially due to complaints of chronic pain, and OT followed with more complications from the surgical wound. There was no difference in terms of hernia recurrence in the short term.

## References

[1] Bhangu A, Fitzgerald JE, Singh P, Battersby N, Marriott P, Pinkney T (2013). Systematic review and meta-analysis of prophylactic mesh
placement for prevention of incisional hernia following midline
laparotomy. Hernia.

[2] Blair LJ, Cox TC, Huntington CR, Groene SA, Prasad T, Lincourt AE (2017). The effect of component separation technique on quality of life
(QOL) and surgical outcomes in complex open ventral hernia repair
(OVHR). Surg Endosc.

[3] Borud LJ, Grunwaldt L, Janz B, Mun E, Slavin SA (2007). Components Separation Combined with Abdominal Wall Plication for
Repair of Large Abdominal Wall Hernias following Bariatric
Surgery. Plast Reconstr Surg.

[4] Burger JWA, Luijendijk RW, Hop WCJ, Halm JA, Verdaasdonk EGG, Jeekel J (2004). Long-term Follow-up of a Randomized Controlled Trial of Suture
Versus Mesh Repair of Incisional Hernia. Ann Surg.

[5] Carbonell II AM, Novitsky YW (2016). Rives-Stoppa Retromuscular Repair. Hernia Surgery.

[6] Chevrel JP, Rath AM (1997). The use of fibrin glues in the surgical treatment of incisional
hernias. Hernia.

[7] Eriksson A, Rosenberg J, Bisgaard T (2014). Surgical treatment for giant incisional hernia a qualitative
systematic review. Hernia.

[8] Gemici K, Acar T, Baris S, Yildiz M, Sever C, Bilgi M (2015). Lower recurrence rate with full-thickness mesh fixation in
incisional hernia repair. Hernia.

[9] Heniford BT, Lincourt AE, Walters AL, Colavita PD, Belyansky I, Kercher KW (2018). Carolinas Comfort Scale as a Measure of Hernia Repair Quality of
Life. Ann Surg.

[10] Heniford BT, Park A, Ramshaw BJ, Voeller G, Hunter JG, Fitzgibbons RJ (2003). Laparoscopic Repair of Ventral Hernias Nine Years' Experience
with 850 Consecutive Hernias. Annals of Surgery.

[11] Heniford BT, Walters AL, Lincourt AE, Novitsky YW, Hope WW, Kercher KW (2008). Comparison of Generic Versus Specific Quality-of-Life Scales for
Mesh Hernia Repairs. J Am Coll Surg.

[12] Kingsnorth A, LeBlanc K (2003). Hernias inguinal and incisional. Lancet.

[13] Klima DA, Tsirline VB, Belyansky I, Dacey KT, Lincourt AE, Kercher KW (2013). Quality of life following component separation versus standard
open ventral hernia repair for large hernias. Surg Innov.

[14] Nassif AT, Nagano TA, Okayama S, Nassif LS, Branco A, Sampaio J (2017). Performance of the Bard Scoring System in Bariatric Surgery
Patients with Nonalcoholic Fatty Liver Disease. Obes Surg.

[15] de Oliveira CM, Nassif AT, B AJ, Nassif LS, Wrubleski T de A, Cavassola AP (2019). Factibility of open vertical gastrectomy in brazil´s public
health system. Rev Col Bras Cir.

[16] Parker SG, Wood CPJ, Butterworth JW, Boulton RW, Plumb AAO, Mallett S (2018). A systematic methodological review of reported perioperative
variables, postoperative outcomes and hernia recurrence from randomised
controlled trials of elective ventral hernia repair clear definitions and
standardised datasets are needed. Hernia.

[17] Petro CC, Novitsky YW (2016). Classification of Hernias. Hernia Surgery.

[18] Porcelli IC de S, Corsi NM, Fracasso M de LC, Pascotto RC, Cardelli AAM, Poli-Frederico RC (2019). Oral health promotion in patients with morbid obesity after
gastroplasty a randomized clinical trial. ABCD Arq Bras Cir Dig (São Paulo).

[19] Rao RS, Gentileschi P, Kini SU (2011). Management of ventral hernias in bariatric
surgery. Surg Obes Relat Dis.

[20] Shell DH, de la Torre J, Andrades P, Vasconez LO (2008). Open Repair of Ventral Incisional Hernias. Surg Clin North Am.

[21] Snyder CW, Graham LA, Gray SH, Vick CC, Hawn MT (2011). Effect of mesh type and position on subsequent abdominal
operations after incisional hernia repair. J Am Coll Surg.

[22] Timmermans L, de Goede B, van Dijk SM, Kleinrensink G-J, Jeekel J, Lange JF (2014). Meta-analysis of sublay versus onlay mesh repair in incisional
hernia surgery. Am J Surg.

[23] Vorst AL, Kaoutzanis C, Carbonell AM, Franz MG (2015). Evolution and advances in laparoscopic ventral and incisional
hernia repair. World J Gastrointest Surg.

